# Surviving critical illness: what is next? An expert consensus statement on physical rehabilitation after hospital discharge

**DOI:** 10.1186/s13054-016-1508-x

**Published:** 2016-10-29

**Authors:** M. E. Major, R. Kwakman, M. E. Kho, B. Connolly, D. McWilliams, L. Denehy, S. Hanekom, S. Patman, R. Gosselink, C. Jones, F. Nollet, D. M. Needham, R. H. H. Engelbert, M. van der Schaaf

**Affiliations:** 1 ACHIEVE—Centre of Applied Research, Faculty of Health, Amsterdam University of Applied Sciences, Amsterdam, The Netherlands; 2 European School of Physiotherapy, Amsterdam University of Applied Sciences, Amsterdam, The Netherlands; 3 McMaster University, School of Rehabilitation Science, Hamilton, Canada; 4 Guy’s & St Thomas’ NHS Foundation Trust and King’s College London, Lane Fox Clinical Respiratory Physiology Research Unit, London, UK; 5 University Hospitals Birmingham NHS Foundation Trust, Queen Elizabeth Hospital Birmingham, Therapy Services, Birmingham, UK; 6 The University of Melbourne, Department of Physiotherapy, Melbourne, Australia; 7 Stellenbosch University, Physiotherapy Division, Department of Interdisciplinary Health Sciences, Faculty of Medicine and Health Sciences, Cape Town, South Africa; 8 The University of Notre Dame Australia, School of Physiotherapy, Fremantle, Australia; 9 KU Leuven – University of Leuven, Department of Rehabilitation Sciences, Leuven, Belgium; 10 University of Liverpool, Musculoskeletal Biology, Institute of Ageing & Chronic Disease, Liverpool, UK; 11 Academic Medical Center, University of Amsterdam, Department of rehabilitation medicine, PO Box 22660, 1100DD Amsterdam, The Netherlands; 12 Johns Hopkins University Baltimore, Outcomes after Critical Illness and Surgery Group, Baltimore, USA; 13 Johns Hopkins University School of Medicine Division of Pulmonary and Critical Care Medicine, Baltimore, USA; 14 Johns Hopkins University School of Medicine Baltimore, Department of Physical Medicine and Rehabilitation, Baltimore, USA

**Keywords:** Consensus statement, Critical illness, Post-intensive care syndrome, Physical therapy, Rehabilitation, Intensive care

## Abstract

**Background:**

The study objective was to obtain consensus on physical therapy (PT) in the rehabilitation of critical illness survivors after hospital discharge. Research questions were: what are PT goals, what are recommended measurement tools, and what constitutes an optimal PT intervention for survivors of critical illness?

**Methods:**

A Delphi consensus study was conducted. Panelists were included based on relevant fields of expertise, years of clinical experience, and publication record. A literature review determined five themes, forming the basis for Delphi round one, which was aimed at generating ideas. Statements were drafted and ranked on a 5-point Likert scale in two additional rounds with the objective to reach consensus. Results were expressed as median and semi-interquartile range, with the consensus threshold set at ≤0.5.

**Results:**

Ten internationally established researchers and clinicians participated in this Delphi panel, with a response rate of 80 %, 100 %, and 100 % across three rounds. Consensus was reached on 88.5 % of the statements, resulting in a framework for PT after hospital discharge. Essential handover information should include information on 15 parameters. A core set of outcomes should test exercise capacity, skeletal muscle strength, function in activities of daily living, mobility, quality of life, and pain. PT interventions should include functional exercises, circuit and endurance training, strengthening exercises for limb and respiratory muscles, education on recovery, and a nutritional component. Screening tools to identify impairments in other health domains and referral to specialists are proposed.

**Conclusions:**

A consensus-based framework for optimal PT after hospital discharge is proposed. Future research should focus on feasibility testing of this framework, developing risk stratification tools and validating core outcome measures for ICU survivors.

**Electronic supplementary material:**

The online version of this article (doi:10.1186/s13054-016-1508-x) contains supplementary material, which is available to authorized users.

## Background

Interdisciplinary interventions directed towards early mobilization of critically ill patients within ICUs are implemented in many hospitals across the world [1, 2]. Serious functional decline associated with immobility, sedation, pharmacological treatment, and mechanical ventilation has been shown in recent publications [3–8]. Long-term impairments in physical and mental health associated with prolonged ICU stay and impeding recovery have now been characterized as post-intensive care syndrome (PICS) [9].

The Society of Critical Care Medicine (SCCM) recommends improvement of continuity of care for ICU survivors, involving risk assessment and comprehensive documentation during all phases of recovery [10]. In the absence of established care pathways or evidence-based guidelines, physical therapists involved in the treatment of patients after hospital discharge conceivably draw on clinical expertise with patients within the cardiopulmonary scope of practice, for which such evidence does exist [11]. However, because the recovery process of survivors of critical illness is explicitly different to the aforesaid group—due to the consequences of critical illness, medical interventions, and persistent systemic inflammation [12]— rehabilitation needs likely extend beyond the physical domain.

The need for standardized sets of outcome measures or a core outcome set (COS) for survivors of critical illness has been highlighted in recent publications [13–16]. A COS aids researchers and clinicians in selecting measurement tools for a certain population. Measuring the core outcomes is essential, while additional measurements can be undertaken dependent on individual patient needs [17]. Currently, no consensus exists on a COS for survivors of critical illness. Several ‘COS for trials’ projects are registered with the Core Outcome Measures in Effectiveness Trials (COMET) initiative [18], but published results are lacking. A ‘COS for clinical practice’ likely differs from a ‘COS for trials’ because instruments used in physical therapy (PT) practice must be practical and feasible, as well as psychometrically solid to contribute to an evidence-based clinical decision-making process [19, 20].

In the absence of scientific evidence, Delphi processes can be used to unite researchers, clinicians, patients, and stakeholders in collaborative initiatives aiming to produce a consensus statement [21]. Results of such studies may contribute to the post-ICU rehabilitation knowledge base, facilitate feasibility studies, and randomized controlled trials (RCTs), and assist in implementing evidence-based interventions across the continuum of care. The aim of this study was to develop, through the use of Delphi methodology, a consensus statement including recommendations for PT practice for survivors of critical illness after hospital discharge. Leading research questions were: what are PT goals, what are recommended measurement tools, and what constitutes an optimal PT intervention for survivors of critical illness?

## Methods

**Fig. 1:**
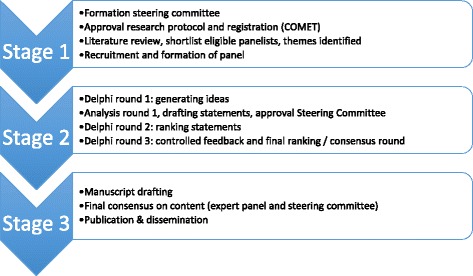
*Delphi Consensus Process.* *COMET* Core Outcome Measures in Effectiveness Trials

The design of this Delphi project consisted of three stages (Fig. 1). An independent steering committee—consisting of experts in the field of rehabilitation medicine, ICU PT, and ICU aftercare at the Academic Medical Center in Amsterdam, the Netherlands—supervised all stages.

A scoping literature review was conducted during March and April 2015 searching PubMed, Medline, PEDro, CINAHL, Science Direct, and ProQuest Social Sciences. Articles were considered for review if they were clinical trials, published in the last 10 years, and PT was the studied intervention. The Appendix illustrates the search strategy. Data were extracted, themes identified, and statements drafted by MEM and RK and approved by the steering committee.

Stage 2 consisted of a three-round Delphi process. A final consensus meeting was not feasible considering the international character of our panel; hence consensus was sought through discussion of the manuscript’s content.

### Panel recruitment

A purposive selected expert panel was used. A shortlist of eligible panelists, derived from the literature review, was approved by the steering committee. Eligibility was determined based on field of expertise and relevant publications indexed in PubMed/Medline. Anonymity of the panelists was assured throughout all Delphi rounds. With acceptance of the invitation, informed consent was obtained for publication of the results.

### Delphi methodology

The need for a minimum of three online Delphi rounds was estimated prior to the start, as per literature recommendations in situations where the quantity of scientific research is limited [21, 22]. In the first round, panelists generated ideas within five themes identified through the literature review (Table 1). Open and closed questions were drafted by MEM and RK. Open questions related to opinions and experiences with PT care after ICU and hospital discharge. Closed questions related to the panelist’s view on the relevance on patient information, measurement tools, and interventions. The answers to the closed questions were dichotomized as either relevant or nonrelevant for PT after hospital discharge. Items unanimously marked as ‘nonrelevant’ were excluded from following rounds. Open question answers were analyzed for transcending themes. Results of round one were formatted into 83 statements, within three categories: hospital phase, hospital discharge information, and post-hospital phase.

**Table 1:** Themes defined for Delphi round one

Theme 1	Defining the patient with PICS. Most common impairments in body functions, structures, activity limitations, and restrictions in participation (International Classification of Functioning, Disability and Health)
Theme 2	Discharge information, which should be made available to the physical therapist after hospital discharge
Theme 3	Reliable and validated outcome measures to use in daily physical therapy practice through the different phases of recovery
Theme 4	Optimal physical therapy interventions
Theme 5	The critical care pathway

*PICS* post-intensive care syndrome

Round two required panelists to rank each statement on an ordinal scale from 1 to 5 (1 = essential, 2 = very important, 3 = important, 4 = unimportant, and 5 = undesirable) [23]. For the third round, each panel member received controlled feedback consisting of group and personal scores (median and semi-interquartile range (SIQR)) for round two. Panelists were asked to re-rank the statements if their individual score lay outside the SIQR. Explanation was required when panelists chose not to adjust their score to the group’s consensus. The Delphi process was terminated once consensus was reached on ≥80 % of the statements, because additional Delphi rounds were not expected to provide potentially different results [21].

### Statistical analysis and consensus

The median and SIQR were calculated for each statement, an appropriate statistical choice for data scored on an ordinal scale [24]. The SIQR was expressed as half the numerical distance between the first and third quarters of the interquartile range (IQR). Consensus was defined a priori as SIQR ≤ 0.5.

The project was registered within the COMET initiative database [25].

## Results

All shortlisted panelists agreed to participate (*N* = 10). The response rate was 80 %, 100 %, and 100 % respectively for the three Delphi rounds. Table 2 presents the countries, disciplines, and field of expertise represented by the panel.

**Table 2:** International Delphi panel characteristics

Number	Country	Field of expertise (title)	Years of clinical experience	Number of publications indexed in PubMed	Agreed to participate	Response		
						Round 1	Round 2	Round 3
1	Australia	Physical therapy (Prof. Dr)	>20	68	√	–	√	√
2	Australia	Physical therapy (Associate Prof. Dr)	>20	12	√	√	√	√
3	Belgium	Physical therapy/movement science (Associate Prof. Dr)	>20	128	√	√	√	√
4	Canada	Physical therapy (Dr)	15–20	44	√	–	√	√
5	The Netherlands	Physical therapy (Associate Prof. Dr)	>20	16	√	√	√	√
6	South Africa	Physical therapy (Associate Prof. Dr)	>20	24	√	√	√	√
7	United Kingdom	Physical therapy (MSc, physical therapist)	10–15	3	√	√	√	√
8	United Kingdom	Nursing/psychology (Dr)	>20	10	√	√	√	√
9	United Kingdom	Physical therapy (Dr)	15–20	9	√	√	√	√
10	USA	Intensive care medicine (Prof. Dr/MD)	15–20	>200	√	√	√	√

– No response

√ Response obtained

Panelists’ comments after round two, related to discharge information and screening tools, initiated the drafting of four additional statements. Consensus was reached on 88.5 % of the statements after round three; no consensus was reached on the ranking of 10 statements (SIQR > 0.5) (Additional file 1: Table S1).

### Hospital phase

The panel consensually ranked the use of valid ADL instruments to establish patients’ functional level at hospital discharge as very important (score: 2; SIQR: 0.5). Consensus was reached on the importance of screening family members for the presence of PICS—family (PICS-F) (score: 3; SIQR: 0.05), but no consensus was achieved on the importance of screening patients for the presence of PICS at hospital discharge (score: 1.25; SIQR: 0.65). Panelists’ explanations related to the absence of validated risk assessment tools for PICS(-F) and disagreement on the preferred timing of this screening (ICU or hospital discharge). No consensus was reached on education of patient and family on PICS at the time of hospital discharge (score: 2; SIQR: 0.65).

### Hospital discharge information

Consensus was reached on the inclusion of 15 items in the hospital discharge information. Items ranked as essential (score: 1) were: premorbid level of functioning (SIQR: 0); physical, mental, and cognitive course of recovery during hospital stay (SIQR: 0); rehabilitation provided and rehabilitation goals (SIQR: 0); and current psychological, cognitive, and physical state (SIQR: 0.5). Items ranked as very important (score: 2) were: severity of illness (SIQR: 0); pre-ICU psychiatric symptoms (SIQR: 0); physiological response to exercise (SIQR: 0); comorbidities (SIQR: 0.15); diagnosed ICU-acquired weakness (ICU-AW) (SIQR: 0.3); delirium whilst in hospital (SIQR: 0.5); ICU and hospital length of stay (LOS) (SIQR: 0.5); and complications during hospital stay (SIQR: 0.5). Items ranked as important (score: 3) were: specific patient and/or family characteristics such as personal and environmental factors (SIQR: 0.5); and days of immobility (SIQR: 0.5). Inclusion of the Acute Physiology and Chronic Health Evaluation (APACHE) score, information on genetic factors, and biomarkers was ranked unimportant (score: 4; SIQR: 0.3). No consensus was reached on the importance of including details on duration of mechanical ventilation, sedation, and surgery in the discharge information (score: 2; SIQR: 0.65). Panelists considered details on mechanical ventilation and sedation to be related to ICU LOS, an easier measure to report at discharge (Additional file 1: Table S1).

### Physical therapy goals after hospital discharge

The panel reached consensus on the following five goals for PT after hospital discharge. Improvement of function in activities of daily living (ADL) and functional exercise capacity were ranked an essential PT goal (score: 1; SIQR: 0 and SIQR: 0.15 respectively). Improvement of skeletal muscle strength and aerobic capacity were ranked very important PT goals (score: 2; SIQR: 0.05 and SIQR: 0.5 respectively), and targeting respiratory muscle strength was ranked an important PT goal (score: 2.75; SIQR: 0.3).

### Core set of outcome measures

#### Exercise capacity and starting exercise intensity: ranking of tools

Consensus was reached on the importance of using both the 6-minute walk test (6MWT) and the 4-meter time walk/gait speed for functional exercise capacity, with a higher ranking for the 6MWT (score: 2; SIQR: 0.05 versus score: 3; SIQR: 0.05). Cycle ergometry testing was ranked important for establishing submaximal exercise capacity (score: 3; SIQR: 0.5). The 2-minute walk test (2MWT) was unanimously ranked as an unimportant tool for measuring exercise capacity after hospital discharge (score: 4; SIQR: 0).

Two methods for determining the starting exercise intensity—with regards to the exercise program—were consensually ranked important (score: 3; SIQR: 0). The first method, commonly practiced in pulmonary rehabilitation [26, 27], recommends to set the starting exercise intensity for walking on a treadmill at 80 % of the average 6MWT speed or 75 % of the peak Incremental Shuttle Walk Test (ISWT) speed. The second method proposes setting starting exercise intensity at 50–70 % of heart rate reserve, combined with a score of 3–4 on the modified Borg scale for perceived exertion. The use of Cardio-Pulmonary Exercise Testing (CPET) to establish starting exercise intensity was consensually ranked unimportant (score: 4; SIQR: 0.25).

No consensus was reached on the use of the ISWT or CPET for testing exercise capacity (score: 2.5 and 3.5 respectively, SIQR: 0.65). Panelists provided comments regarding the feasibility and practical applicability (CPET) and the lack of data on validity (ISWT) of these measures.

#### Physical functioning: ranking of tools

The following physical function and mobility scales were ranked important (score: 3) in consensus: the De Morton Mobility Index (DEMMI) (SIQR: 0); the Timed Up and Go test (SIQR: 0.15); the Functional Independence Measure (SIQR: 0.15); the Short Physical Performance Battery (SIQR: 0.15); and the Short Form 36—physical function domain (SIQR: 0.5). Consensus was also reached on tools to assess (instrumental) ADL function; the Barthel Index, the KATZ-ADL, and Lawton’s iADL were ranked important (score: 3; SIQR: 0.15).

#### Muscle, nerve integrity, and body composition: ranking of tools

Consensus was reached on the importance of using handgrip (HG) strength and handheld dynamometry (HHD) to establish overall muscle strength, with a higher rating for HG strength (score: 2.25; SIQR: 0.3 versus score: 3; SIQR: 0.05).

Both maximum inspiratory pressure (MIP) and maximum expiratory pressure (MEP) were consensually ranked important tools for measuring respiratory muscle function (score: 3; SIQR: 0 and 0.25 respectively). Consensus was also reached on the importance of using the Medical Research Council (MRC) dyspnea scale (score: 2.5; SIQR: 0.3) for perceived respiratory disability and spirometry (score: 3; SIQR: 0) for pulmonary function.

Ultrasound of large skeletal muscles and anthropometry were ranked important (score: 3) in consensus (SIQR: 0.15), while body composition tests using bio-impedance spectroscopy or multifrequency bio-impedance analysis achieved consensual ranking as unimportant (score: 4; SIQR: 0.15). Nerve conduction studies and electromyography were unanimously and consensually ranked unimportant for usage after hospital discharge (score: 4: SIQR: 0).

No consensus was reached on the importance of using the MRC Sum Score (MRC-SS) for muscle strength, nor for peak expiratory flow measurement after hospital discharge (score: 2.5 and 3.0; SIQR: 0.65).

#### Quality of life and pain: ranking of tools

The Short Form 36 and the EuroQol^©^ Health Questionnaire (EQ-5D) were consensually ranked as very important (score: 2; SIQR: 0 and 0.5 respectively), and both were ranked higher than the Sickness Impact Profile (score: 3; SIQR: 0.15). The visual analogue scale (VAS) for pain was unanimously ranked as very important (score: 2; SIQR: 0) (Additional file 1: Table S1).

### Physical therapy interventions

Consensus was achieved in ranking functional exercises (score: 1.25; SIQR: 0.5), circuit training and endurance training (both score: 2; SIQR: 0.15), and range of motion exercises and balance training (both score: 2; SIQR: 0.5) as very important PT interventions for improving physical function in survivors of critical illness after hospital discharge. Interval training (SIQR: 0) and high-intensity interval training (SIQR: 0.3) were both consensually ranked important (score: 3).

Targeting muscle strength through strengthening exercises and nutritional support achieved consensual ranking as very important interventions (score: 1.5; SIQR: 0.25 and 0.45 respectively). Inspiratory and expiratory muscle training consensually ranked 3.5 (SIQR 0.3 and 0.5 respectively), suggestive of being useful additional interventions, dependent on assessment outcomes. Neuromuscular electrical stimulation (NMES) achieved consensual ranking of unimportant (score: 4; SIQR: 0) as a PT intervention after hospital discharge.

Education of patients and caregiver(s) on PICS as well as involvement of caregivers in the rehabilitation process was unanimously ranked as an essential PT intervention after hospital discharge (score: 1; SIQR: 0). No consensus was reached on the importance of relaxation exercises (score: 2; SIQR: 0.65).

### Other health domains: ranking of screening tools

From a predefined list of screening tools for other PICS-related impairments, panelists consensually ranked the Multidimensional Fatigue Inventory (MFI) or the modified Borg scale for the presence of fatigue as very important (score: 2; SIQR: 0.05). The Hospital Anxiety and Depression Scale was ranked as very important (score: 2; SIQR: 0.25) and the Impacts of Events Scale—Revised as important (score: 3; SIQR: 0) for screening for problems in the psychological domain. The Mini Mental State Examination for cognitive function, the Subjective Global Assessment Tool, Malnutrition Universal Screening Tool, or Short Nutritional Assessment Questionnaire for nutritional status, and the Richard Campbell Sleep Questionnaire for sleep quality were all ranked important (score: 3; SIQR: 0) in consensus. No consensus was reached on the importance of the Trauma Screening Questionnaire for post-traumatic stress syndrome (PTSS) (score: 3; SIQR: 0.65).

## Discussion

**Fig. 2:**
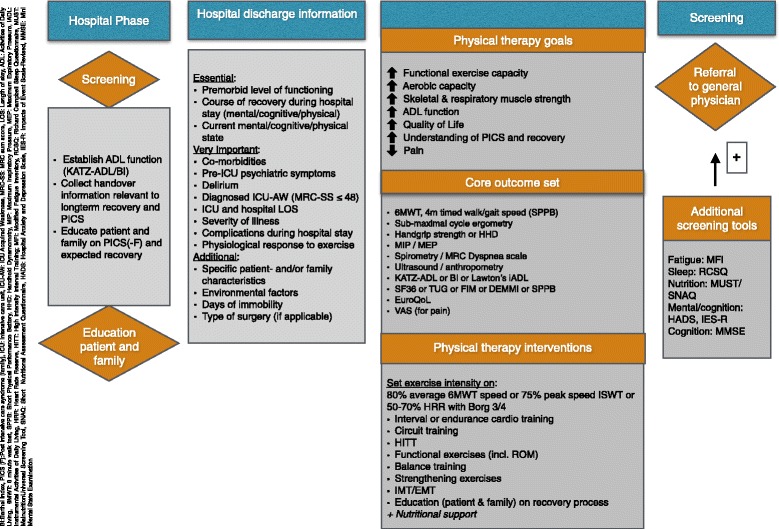
Physical therapy after critical illness: a consensus-based framework

This Delphi project resulted in consensus rankings of statements related to PT goals, a COS, and PT interventions for survivors of critical illness after hospital discharge. An international panel of ICU rehabilitation experts rated the importance of each statement on a 5-point scale, with scores from 1 = ‘essential’ to 5 = ‘undesirable’. We propose the use of a consensus-based framework to optimize the transition and recovery of critical illness survivors after hospital discharge. This framework contains recommendations for essential discharge information, PT goals, a COS, and optimal PT interventions (Fig. 2).

Although critical illness survivors might seek PT without referral in countries with direct access [7, 8], a formal and structured care pathway may more appropriately address patients’ comprehensive rehabilitation needs [10]. Initiatives such as multidisciplinary follow-up clinics succeed in assessing recovery problems in patients after hospital discharge [28, 29], but do not offer rehabilitation interventions. Additionally, follow up often commences only after 3 months, consequently not utilizing the time window of recovery directly after discharge [30]. Our framework aims to facilitate a continuum of rehabilitation across all phases of post-ICU recovery.

Risk assessment for the development of PICS and PICS-F at hospital discharge was a topic of discussion within the panel. Although ranked essential, consensus was not achieved on the importance of screening patients for PICS at hospital discharge. This could be explained by the phrasing of the statement, because it implied the presence of a valid screening tool (Additional file 1: Table S1). Priority should be given to the development and validation of a risk assessment tool to facilitate optimal rehabilitation pathways for individual patients. Promising results in recent publications clarify patient-specific, ICU-specific, and environmental-specific factors affecting long-term outcomes [31–34]. Risk stratification based on pre-existing chronic disease, ICU LOS, or age might predict recovery outcomes and health care usage and may assist in determining tailor-made rehabilitation interventions within this proposed framework [33, 34].

This study resulted in a consensus statement on essential handover information at the time of hospital discharge. Fifteen parameters related to critical illness and recovery, as well as known risk factors for PICS [10], were ranked very high in importance. Currently these data are rarely provided in discharge summaries [35] and further testing should determine the feasibility of collecting these data at hospital discharge.

This Delphi process resulted in consensus on PT goals and interventions for critical illness survivors after hospital discharge. Exercise programs should target the cardiovascular system, as well as skeletal muscle strength, range of motion (ROM), balance, and function in ADL, dependent on the outcome of the assessment. Two methods for setting exercise intensity are proposed, with no preference for one over the other. Although respiratory muscle training was consensually ranked an important PT intervention, panelists commented on the lack of evidence for effectiveness in this population after hospital discharge. The panel consensually ranked additional nutritional support as very important. A combined exercise and nutrition intervention was not addressed in this Delphi project but a recently published RCT showed positive effects of such an intervention on walking distance at 3 months [36].

Reaching consensus on a core set of outcomes proved difficult. Quality-of-life scales, the VAS scale for pain, HG strength, and the 6MWT were the only tools scoring ‘very important’ in consensus. The 6MWT is a widely used test, is feasible, and is validated for the population of ICU survivors [14, 37, 38]. Disadvantages could be the expected ceiling effects with patients who have greater initial cardiovascular fitness or in later phases of recovery. Criterion validity has not so far been established [39]. Predicting maximum exercise capacity by means of the ISWT may be an appropriate alternative, because criterion validity against the CPET was established [40–42] and psychometric properties of the ISWT in similar populations yield promising results. This Delphi panel, however, did not reach consensus on the usage of the ISWT after hospital discharge.

Several mobility scales were ranked ‘important’ but many have yet to be validated for this population in the post-hospital situation. Such a tool could be the DEMMI, for which psychometric properties were recently established for survivors of critical illness, albeit within the hospital setting [43].

Screening for PICS-related cognitive and mental impairments is deemed essential for establishing an optimal rehabilitation pathway, because these factors potentially influence the outcome of rehabilitation interventions. Dependent on country and setting, physical therapists can assist in screening and refer to specialist health professionals when such screenings are not conducted at ICU-follow-up clinics [7, 8, 29, 30].

This consensus statement complements published evidence statements on safe and effective PT interventions in the ICU [1, 2, 23], and contributes to the provision of optimal PT throughout the continuum of care, from critical illness to full participation and return to work.

### Limitations to this study

Although eligible panelists were carefully recruited, selection bias could not be prevented. The panel included a heterogeneous group of researchers and clinicians from different countries, settings, and cultural backgrounds. Although this heterogeneity might strengthen the consensus statement and its practical applicability worldwide, it is emphasized that the results of this Delphi study should be seen as an adjustable framework rather than as a directive guideline.

The small sample size and the absence of survivors of critical illness or caregivers in this expert panel are limitations to this study because important input from other perspectives is lacking.

The 5-point Likert scale is a commonly used ranking scale in Delphi procedures [23]. Although the ordinal scale was carefully explained to the panel, it was considered likely that panelists would select ‘important’ (score: 3) in cases where they felt indifferent to a certain item. This scoring possibly affected the outcome of rounds two and three. Future Delphi projects should clarify this 5-point Likert scale or consider a 9-point ranking scale. It should also be noted that scoring related to ‘relevance’ rather than practicality and feasibility in clinical practice, which necessitates feasibility testing of the proposed framework.

### Recommendations for future research

Future Delphi panels should include a larger group of representatives from a variety of health disciplines as well as survivors of critical illness to incorporate all health domains relevant to rehabilitation of critically ill patients.

Efforts on development and validation of a screening tool for PICS should continue to be a research priority in order to determine patients’ rehabilitation needs and design tailor-made interventions.

Psychometric properties of the proposed core outcome measures for out-of-hospital PT practice should be established for the population of critical illness survivors.

Within the proposed framework for PT interventions after hospital discharge, feasibility studies and RCTs must be set up to investigate intervention effectiveness and appropriateness of exercise training modalities.

## Conclusions

This consensus-based framework for PT after hospital discharge aims to improve long-term outcomes for survivors of critical illness. Physical therapists should seek close collaboration with the multidisciplinary team at ICU-follow-up clinics (when available) when assessing rehabilitation needs. Multimodal and targeted exercise interventions should be set up and feasibility tested. Future research should focus on validation of core measurement tools for cognitive, mental, and physical function in the population of critical illness survivors at different points of their recovery trajectory.

## Appendix

Search strategy scoping review

**Table:**

Database	Search terms	Hits	For review
PubMed	#1 “Critical Care”[Mesh] OR “Intensive Care”[Mesh] AND “Rehabilitation”[Mesh] OR “Aftercare”[Mesh]	7017	
PubMed	#2 “Critical Care”[Mesh] OR “Intensive Care”[Mesh] AND “Rehabilitation”[Mesh] AND “Aftercare”[Mesh]	9	2
PubMed	#3 “Critical Care”[Mesh] OR “Intensive Care”[Mesh] AND “Aftercare”[Mesh] (Limits: last 10 years, clinical trials only; Filters: adults)	115	3
PubMed	#4 post intensive care syndrome[Title/Abstract]) OR PICS[Title/Abstract] (Limits: adults 19+ published last 10 years)	53	1
PubMed	#5 intensive care[Title/Abstract] OR ICU[Title/Abstract] AND survivor*[Title/Abstract] (Limits: adult/last 10 years/clinical trial)	227	13
PubMed	#6 intensive care[Title/Abstract] OR ICU[Title/Abstract] AND surviv*[Title/Abstract] AND recovery[Title/Abstract] (Limits: adults 19+, last 10 years, clinical trials)	51	6
PubMed	#7 “Critical Care”[Mesh] OR “Intensive Care”[Mesh] AND “Rehabilitation”[Mesh] AND after care	10	0
PubMed	#8 “Critical Care”[Mesh] OR “Intensive Care”[Mesh] AND “Rehabilitation”[Mesh] (Limits last 10 years, adults 19+)	103	16
PEDro	#1 Critical care	129	3
CINAHL	#1 AB Critical Care AND AB rehabilitation	137	16
CINAHL	#2 AB Critical Care AND physical therapy OR physiotherapy AND recovery (Limits: last 10 years, all adult)	116	0
CINAHL	#3 post intensive care syndrome [Title/Abstract]	12	6
Medline	#1 Critical Care AND post intensive care syndrome	8	8
Medline	#2 Post intensive care syndrome	14	7
Science Direct	#1 Critical Care (title/abstract/key words) Rehabilitation (title/abstract/key words) #2 pub-date > 2003 and TITLE (post intensive care syndrome) or TITLE-ABSTR-KEY (post intensive care syndrome) #3 pub-date > 2003 and TITLE (post intensive care syndrome) or TITLE-ABSTR-KEY (post intensive care syndrome) AND LIMIT-TO(topics, “icu”)	203 293 12	9
ProQuest Social Sciences	#1 SU.EXACT (“Intensive care”) AND SU.EXACT(“Rehabilitation”) OR SU.EXACT(“After care”) 2004–2015 #2 Limits: peer reviewed #3 Limits: rehabilitation	232 187 20	1
PubMed (9 April 2015)	#1 (“Critical Care”[Mesh]) OR “Critical Illness”[Mesh] AND “Physical Therapy Modalities”[Mesh] OR “Exercise”[Mesh] OR “Exercise Therapy”[Mesh] OR “Physical Therapy Specialty”[Mesh]) #2 (“Critical Care”[Mesh] OR “Critical Illness”[Mesh] AND “Physical Therapy Modalities”[Mesh] OR “Exercise”[Mesh] OR “Exercise Therapy”[Mesh] OR “Physical Therapy Specialty”[Mesh] AND Humans[Mesh] AND adult[MeSH] AND recovery[Title/Abstract] OR post intensive care[Title/Abstract]) #3 (“Intensive Care”[Mesh] AND “Critical Care”[Mesh] OR “Critical Illness”[Mesh] AND “Rehabilitation”[Mesh] OR “Aftercare”[Mesh] AND “Physical Therapy Modalities”[Mesh]) limits last 10 years, adults #4 (“Intensive Care”[Mesh] AND “Critical Care”[Mesh] OR “Critical Illness”[Mesh] AND “Physical Therapy Modalities”[Mesh]) limits: last 10 years, adults	74,431 5413 33 85	3 (duplicates) 7 (6 duplicates)
PubMed (diagnostics)	#1 (“Diagnosis”[Mesh] AND “Intensive Care”[Mesh] OR “Critical Illness”[Mesh] AND rehabilitation[Title/Abstract] OR physical therapy modalities[MeSH Terms] AND recovery[Title/Abstract]) #2 (“Exercise Tolerance”[Mesh] OR “Exercise Therapy”[Mesh] OR “Exercise”[Mesh] AND “Survivors”[Mesh] AND “Critical Illness”[Mesh]) #3 (“Exercise Tolerance”[Mesh] OR “Exercise Therapy”[Mesh] OR “Exercise”[Mesh] AND “Survivors”[Mesh] OR “Critical Illness”[Mesh]) #4 (“Muscle Strength”[Mesh] AND “Survivors”[Mesh] AND “Critical Illness”[Mesh]) #5 (“Exercise Test”[Mesh] AND “Critical Illness”[Mesh]) #6 (“Outcome Assessment (Health Care)”[Mesh] AND “Critical Illness”[Mesh] AND survivor*[Title/Abstract])	4466 4 5271 1 15 104	1 1 1 36

Searches were done on 13 March 2015, 19 March 2015, 26 March 2015, and 9 April 2015; alerts were entered into the appropriate database with similar search terms

## Additional file

Additional file 1: is Table S1 presenting statements and ranking Delphi rounds two and three. (DOC 127 kb)

## Abbreviations

2MWT: 2-Minute walk test
6MWT: 6-Minute walk test
ADL: Activities of daily living
APACHE: Acute Physiology and Chronic Health Evaluation
COMET: Core Outcome Measures in Effectiveness Trials
COS: Core outcome set
CPET: Cardio-pulmonary Exercise Testing
DEMMI: de Morton Mobility Index
EQ-5D: EuroQol 5 Dimensions Health Questionnaire
HG: Hand grip
HHD: Hand-held dynamometer
iADL: Instrumental activities of daily living
ICF: International Classification of Functioning, Disability and Health
ICU: Intensive care unit
ICU-AW: Intensive care unit-acquired weakness
ISWT: Incremental Shuttle Walk Test
LOS: Length of stay
MEP: Maximum expiratory pressure
MFI: Modified fatigue inventory
MIP: Maximum inspiratory pressure
MRC: Medical Research Council
MRC-SS: Medical Research Council Sum Score
PICS: Post-intensive care syndrome
PICS-F: Post-intensive care syndrome—family
PT: Physical therapy
PTSS: Post-traumatic stress syndrome
RCT: Randomized controlled trial
ROM: Range of motion
SCCM: Society of Critical Care Medicine
SIQR: Semi-interquartile range
VAS: Visual analogue scale
