# Supplementary material for: Surviving critical illness: what is next? An expert consensus statement on physical rehabilitation after hospital discharge
Source: Crit Care. 2016 Oct 29;20:354. doi: 10.1186/s13054-016-1508-x (PMC5086052; doi:10.1186/s13054-016-1508-x)
Supplement: Additional file 1: — is Table S1 presenting statements and ranking Delphi rounds two and three. (DOC 127 kb) [file 13054_2016_1508_MOESM1_ESM.doc]

***Table S1***: Statements and ranking Delphi round 2 and 3

| Statement | Round 2  Median (SIQR) | Round 3  Median (SIQR) | Consensus |
| --- | --- | --- | --- |
| **Hospital Phase** |  |  |  |
| The patient is discharged from hospital when his (independent) functional level is established (through KATZ-ADL, Barthel Index or similar measure) and his home environment is carefully analyzed | 2.0 (0.65) | 2.0 (0.5) | ✔︎ |
| At the time of discharge from hospital the patient’s family should be screened for the presence (or risk of development) of PICS-F | 3.0 (0.5) | 3.0 (0.05) | ✔︎ |
| At the time of discharge from hospital the patient should be screened for the presence (or risk of development) of PICS | 1.5 (0.65) | 1.25 (0.65) | ✗ |
| At the time of discharge from the hospital the patient and family should be educated on PICS and expected recovery (i.e. rehab manual) | 2.0 (0.65) | 2.0 (0.65) | ✗ |
| **Essential discharge information** |  |  |  |
| Course of recovery of critical illness during hospital stay (mental, cognitive, physical) | 1.0 (0.5) | 1.0 (0) | ✔︎ * |
| Rehabilitation provided, goals achieved and further rehabilitation goals | 1.0 (0.15) | 1.0 (0) | ✔︎ * |
| Premorbid level of functioning | 1.0 (0.65) | 1.0 (0) | ✔︎ |
| Current psychological state (i.e. presence of depression) | ** | 1.0 (0.5) | ✔︎ |
| Current cognitive functioning | ** | 1.0 (0.5) | ✔︎ |
| Current physical functioning | ** | 1.0 (0.5) | ✔︎ |
| Diagnosed ICU-Acquired Weakness (MRC score) | 1.5 (1.0) | 1.5 (0.3) | ✔︎ |
| Pre-ICU psychiatric symptoms | 2.0 (0.65) | 2.0 (0) | ✔︎ |
| Physiological response to exercise | 2.0 (1.0) | 2.0 (0) | ✔︎ |
| Severity of illness (i.e. sepsis / multi organ failure / other) | 2.0 (0.5) | 2.0 (0) | ✔︎ |
| Comorbidities | 2.0 (1.0) | 2.0 (0.15) | ✔︎ |
| ICU length of stay | 2.0 (0.5) | 2.0 (0.5) | ✔︎ |
| Hospital length of stay | 2.0 (0.65) | 2.0 (0.5) | ✔︎ |
| Presence of delirium while in hospital | 2.0 (0.65) | 2.0 (0.5) | ✔︎ |
| Complications during hospital stay | 2.0 (1.0) | 2.0 (0.5) | ✔︎ |
| Specific patient- and/or family characteristics (personal factors / environmental factors) | 3.0 (0.5) | 3.0 (0.5) | ✔︎ |
| Days of immobility (defined as being bed-bound) | 3.0 (0.65) | 3.0 (0.5) | ✔︎ |
| Apache II or Apache IV score | 3.5 (0.5) | 3.5 (0.3) | ✔︎ |
| Genetic factors / biomarkers | 3.5 (0.5) | 3.5 (0.3) | ✔︎ |
| Days of sedation and mechanical ventilation | 2.0 (1.0) | 2.0 (0.65) | ✗ |
| Surgery | 2.0 (1.0) | 2.0 (0.65) | ✗ |
| **Post-discharge phase: physical therapy goals** |  |  |  |
| Improvement of physical function and function in ADL | 1.0 (0.0) | 1.0 (0) | ✔︎ |
| Improvement of functional exercise capacity | 1.0 (0.65) | 1.0 (0.15) | ✔︎ |
| Improvement of skeletal muscle strength | 2.0 (1.0) | 2.0 (0.05) | ✔︎ |
| Improvement of aerobic capacity | 2.0 (0.65) | 2.0 (0.5) | ✔︎ |
| Improvement of respiratory muscle strength | 2.5 (1.0) | 2.75 (0.3) | ✔︎ |
| **Core outcome measures after hospital discharge** |  |  |  |
| Pain (such as joint pain) should be measured through the use of the Visual Analogue Scale (VAS) | 2.0 (1.3) | 2.0 (0) | ✔︎ |
| Quality of life should be measured through the use of the Short Form 36 | 2.0 (1.15) | 2.0 (0) | ✔︎ |
| Functional exercise capacity should be measured through the use of the 6 minute walk test (6MWT) | 2.0 (0.15) | 2.0 (0.05) | ✔︎ |
| Quality of life should be measured through the use of the EuroQol Health Questionnaire | 2.0 (1.5) | 2.0 (0.5) | ✔︎ |
| Skeletal muscle strength should be measured through the use of handgrip strength | 2.5 (0.65) | 2.25 (0.3) | ✔︎ |
| Respiratory muscle function should be measured through the use of MRC dyspnea scale | 2.5 (0.5) | 2.5 (0.3) | ✔︎ |
| Physical function should be measured through the use of the De Morton Mobility Index (DEMMI) | 3.0 (0.25) | 3.0 (0) | ✔︎ |
| Respiratory muscle function should be measured through the use of maximum inspiratory pressure (MIP) | 3.0 (0.65) | 3.0 (0) | ✔︎ |
| Respiratory muscle function should be measured through the use of spirometry | 3.0 (0.5) | 3.0 (0) | ✔︎ |
| Functional exercise capacity should be measured through the use of the 4 m timed walk/gait speed (single test from SPPB) | 3.0 (0.25) | 3.0 (0.05) | ✔︎ |
| Skeletal muscle strength should be measured through the use of handheld dynamometry | 3.0 (0.65) | 3.0 (0.05) | ✔︎ |
| Physical function should be measured through the use of the Timed Up and Go (TUG) | 3.0 (0.5) | 3.0 (0.15) | ✔︎ |
| Physical function should be measured through the use of the Functional Independent Measure (FIM) | 3.0 (0.25) | 3.0 (0.15) | ✔︎ |
| Physical function should be measured through the use of the Short Physical Performance Battery (SPPB) | 3.0 (1.0) | 3.0 (0.15) | ✔︎ |
| Function in ADL should be measured through the use of the Barthel Index | 3.0 (1.0) | 3.0 (0.15) | ✔︎ |
| Function in ADL should be measured through the use of the KATZ-ADL | 3.0 (0.25) | 3.0 (0.15) | ✔︎ |
| Function in ADL should be measured through the use of the Lawton’s iADL | 3.0 (0.25) | 3.0 (0.15) | ✔︎ |
| Body composition (such as muscle mass and fat free mass) should be measured through the use of ultrasound | 3.0 (1.0) | 3.0 (0.15) | ✔︎ |
| Body composition (such as muscle mass and fat free mass should be measured through the use of anthropometry | 3.0 (0.65) | 3.0 (0.15) | ✔︎ |
| Quality of life should be measured through the use of the Sickness Impact Profile 68 | 3.0 (0.65) | 3.0 (0.15) | ✔︎ |
| Respiratory muscle function should be measured through the use of maximum expiratory pressure (MEP) | 3.5 (0.5) | 3.0 (0.25) | ✔︎ |
| Aerobic capacity should be measured through the use of submaximal cycle ergometry test | 3.0 (0.65) | 3.0 (0.5) | ✔︎ |
| Physical function should be measured through the use of short form 36 – physical domain (SF 36-PD) | 3.0 (1.0) | 3.0 (0.5) | ✔︎ |
| Physical function should be measured through the use of the Functional Assessment Measure (FAM) | 3.5 (0.65) | 3.25 (0.3) | ✔︎ |
| Functional exercise capacity should be measured through the use of the 2 minute walk test (2MWT) | 4.0 (0.75) | 4.0 (0) | ✔︎ |
| Neuromuscular function should be measured through the use of electromyography | 4.0 (0.65) | 4.0 (0) | ✔︎ |
| Neuromuscular function should be measured through the use of nerve conduction velocity test | 4.0 (0.65) | 4.0 (0) | ✔︎ |
| Body composition (such as muscle mass and fat free mass) should be measured through the use of bioimpedance spectroscopy (BIS) or multifrequency bioimpedance analysis (BIA) | 4.0 (0.65) | 4.0 (0.15) | ✔︎ |
| Functional exercise capacity should be measured through the use of the incremental shuttle walk test (ISWT) | 2.5 (0.65) | 2.5 (0.65) | ✗ |
| Skeletal muscle strength should be measured through the use of MRC sum score | 2.5 (0.65) | 2.5 (0.65) | ✗ |
| Respiratory muscle function should be measured through the use of peak expiratory flow | 3.0 (0.65) | 3.0 (0.65) | ✗ |
| Aerobic capacity should be measured through the use of cardio-pulmonary exercise testing (CPET) | 3.5 (0.65) | 3.75 (0.65) | ✗ |
| **Post-discharge phase: starting exercise intensity** |  |  |  |
| Starting exercise intensity or aerobic capacity should be set at 80% of the average 6MWT speed or 75% of peak speed achieved on the ISWT (as with formal rehab tracks set for pulmonary rehab) | 3.0 (0.5) | 3.0 (0) | ✔︎ |
| Starting exercise intensity should be set at 50-70% of HR reserve (Karvonen) in combination with the modified Borg 3 or 4 for perceived exertion | 3.0 (0.65) | 3.0 (0) | ✔︎ |
| Starting exercise intensity should be set at 50-80% of VO2max as measured with CPET | 4.0 (1.15) | 4.0 (0.25) | ✔︎ |
| **Post-discharge phase: physical therapy interventions (physical function)** |  |  |  |
| Functional exercises | 1.5 (0.5) | 1.25 (0.5) | ✔︎ |
| Circuit training | 2.0 (0.5) | 2.0 (0.15) | ✔︎ |
| Endurance cardio training | 2.0 (0.65) | 2.0 (0.15) | ✔︎ |
| Balance training | 2.0 (1.0) | 2.0 (0.5) | ✔︎ |
| ROM exercises | 2.5 (0.65) | 2.75 (0.5) | ✔︎ |
| High intensity interval training (HIIT) | 3.0 (0.75) | 3.0 (0) | ✔︎ |
| Interval cardio training | 2.0 (1.0) | 3.0 (0.3) | ✔︎ |
| Relaxation exercises | 2.0 (0.75) | 2.0 (0.65) | ✗ |
| **Physical therapy interventions (skeletal muscle strength)** |  |  |  |
| Strengthening exercises | 1.5 (0.65) | 1.75 (0.25) | ✔︎ |
| Nutritional support | 1.5 (1.0) | 1.75 (0.45) | ✔︎ |
| Inspiratory muscle training | 3.5 (1.0) | 3.0 (0.3) | ✔︎ |
| Expiratory muscle training | 3.5 (1.0) | 3.25 (0.5) | ✔︎ |
| Neuromuscular electrical stimulation (NMES) | 4.0 (0.5) | 4.0 (0) | ✔︎ |
| **Physical therapy interventions (education)** |  |  |  |
| Physical therapists should educate caregivers and patients on the recovery process and PICS in general | 1.0 (0.15) | 1.0 (0) | ✔︎ |
| Physical therapists should involve caregivers in the rehabilitation process of the patient with PICS | 1.0 (0.5) | 1.0 (0) | ✔︎ |
| **Screening tools and referral** |  |  |  |
| Multidimensional Fatigue Inventory or BORG scale for the presence of fatigue | 2.0 (0.65) | 2.0 (0.05) | ✔︎ |
| Mini Mental State Examination for cognitive function | 3.0 (0.65) | 3.0 (0) | ✔︎ |
| Impacts of Events Scale – Revised for psychological well-being and PTSD | 3.0 (0.5) | 3.0 (0) | ✔︎ |
| Subjective Global Assessment Tool, Malnutrition Universal Screening Tool (MUST) or Short Nutritional Assessment Questionnaire (SNAQ) for nutritional status | 3.0 (0.65) | 3.0 (0) | ✔︎ |
| Richard Campbell Sleep Questionnaire for sleep quality | 3.0 (1.0) | 3.0 (0.5) | ✔︎ |
| Trauma Screening Questionnaire for PTSS | ** | 3.0 (0.65) | ✗ |

*Score: 1 = essential, 2 = very important, 3 = important, 4 = unimportant, 5 = undesirable*

*︎︎*✔ *= consensus threshold reached (SIQR ≤ 0.5)* ✗*= no consensus reached * = unanimous score by panel ** = generated idea in round 2, ranked only in round*
